# Fluorescent markers of various organelles in the wheat pathogen *Zymoseptoria tritici*

**DOI:** 10.1016/j.fgb.2017.05.001

**Published:** 2017-08

**Authors:** S. Kilaru, M. Schuster, W. Ma, G. Steinberg

**Affiliations:** a School of Biosciences, University of Exeter, Exeter EX4 4QD, UK; b University of Utrecht, Department of Biology, Padualaan 8, 3584 CH Utrecht, The Netherlands

**Keywords:** Tub2, α-tubulin, eGFP, enhanced green-fluorescent protein, Zt, *Zymoseptoria tritici*, ZtGFP, *Z. tritici* codon-optimised GFP, *sdi1*, succinate dehydrogenase 1, RB and LB, right and left border, mCherry, monomeric cherry, *hph*, hygromycin phosphotransferase, *nptII*, neomycin phosphotransferase, *bar*, phosphinothricin acetyltransferase, STB, Septoria tritici blotch, ER, endoplasmic reticulum, F-actin, Plasma membrane, Autophagosomes, Endoplasmic reticulum, Nuclei, Peroxisome, Septoria tritici blotch in wheat

## Abstract

Development of novel strategies to control fungal plant pathogens requires understanding of their cellular organisation and biology. Live cell imaging of fluorescent organelle markers has provided valuable insight into various aspects of their cell biology, including invasion strategies in plant pathogenic fungi. Here, we introduce a set of 17 vectors that encode fluorescent markers to visualize the plasma membrane, endoplasmic reticulum (ER), chromosomes, the actin cytoskeleton, peroxisomes and autophagosomes in the wheat pathogen *Zymoseptoria tritici*. We fused either enhanced green-fluorescent protein (eGFP) or a codon-optimised version of GFP (ZtGFP) to homologues of a plasma membrane-located Sso1-like syntaxin, an ER signalling and retention peptide, a histone H1 homologue, the LifeAct actin-binding peptide, a mitochondrial acetyl-CoA dehydrogenase, a peroxisomal import signal and a homologue of the ubiquitin-like autophagosomal protein Atg8. We expressed these markers in wildtype strain IPO323 and confirmed the specificity of these markers by counterstaining or physiological experiments. This new set of molecular tools will help understanding the cell biology of the wheat pathogen *Z. tritici*.

## Highlights

• 17 vectors are described that allow labelling of 7 subcellular structures.
• The fluorescent markers target the plasma membrane, endoplasmic reticulum, nucleus.
• Markers also target the actin cytoskeleton, peroxisomes and autophagosomes.
• These markers complete are toolkit of fluorescent reporters.
• Reporters allow cell biological studies in the Septoria tritici blotch fungus.

## 1 Introduction

A growing world population requires more efficient production of food crops, of which wheat is the second most important calorie crop worldwide. World trade in this commodity is greater than that for all other crops combined (Curtis, 2002). Amongst all pest and pathogens, the fungi pose the most serious challenge to food security (Fisher et al., 2012). For example, the fungal pathogen *Zymoseptoria tritici*, the causal agent of Septoria tritici blotch (STB) on wheat, causes economic damage worth ∼720–1440 million Euros/year, despite fungicide treatment of wheat, with chemicals worth ∼930 million Euros, in France, Germany and the United Kingdom alone (Fones and Gurr, 2015). Although *Z. tritici* poses a significant challenge to food security, very little is known about the cellular biology underpinning growth and morphogenesis, or the infection strategy in this fungus (Steinberg, 2015). To address this shortcoming, the British Biotechnology and Biological Sciences Research Council have recently supported extensive research, to develop new tools to study *Z. tritici*. This led to the publication of a special issue in the journal Fungal Genetics and Biology (2015, Volume 79), summarising molecular tools and techniques to study both, *Z. tritici* and the molecular basis of STB infection. Amongst these tools is a codon-optimised green fluorescent protein (ZtGFP, Kilaru et al., 2015c) and various red-fluorescent proteins (Schuster et al., 2015a). These tags were used to develop fluorescent reporters for live cell imaging of various organelles and sub-cellular structures. This collection includes markers for various compartments of the endocytic pathway (Kilaru et al., 2015b), for cell polarity (Guo et al., 2015) and markers that enable observation of the microtubule cytoskeleton (Schuster et al., 2015b). Here we extend this list by additional fluorescent reporters for 7 additional cellular compartments. We report 17 new plasmids, generated by yeast recombination–based techniques (Kilaru and Steinberg, 2015), that label the plasma membrane, nuclei, endoplasmic reticulum, mitochondria, F-actin, peroxisomes and autophagosomes in *Z. tritici*. We integrated these markers into *Z. tritici* by *Agrobacterium tumifaciens*-based transformation and used co-staining with dyes and pharmacological, as well as physiological experiments to confirm the specificity of the fluorescent reporter proteins for sub-cellular structures. These new fluorescent markers provide a significant advancement over the current toolkit for studying *Z. tritici*. This increases the capacity to analyse mutant phenotypes or cellular dynamics of the wheat pathogen during plant infection. Such experiments promise to open new avenues to develop novel ways to control one of the most important fungal pathogen on wheat.

## 2 Results and discussion

### 2.1 Identification of ZtSso1, ZtHis1, ZtAcd1 and ZtAtg8

**Fig. 1:**
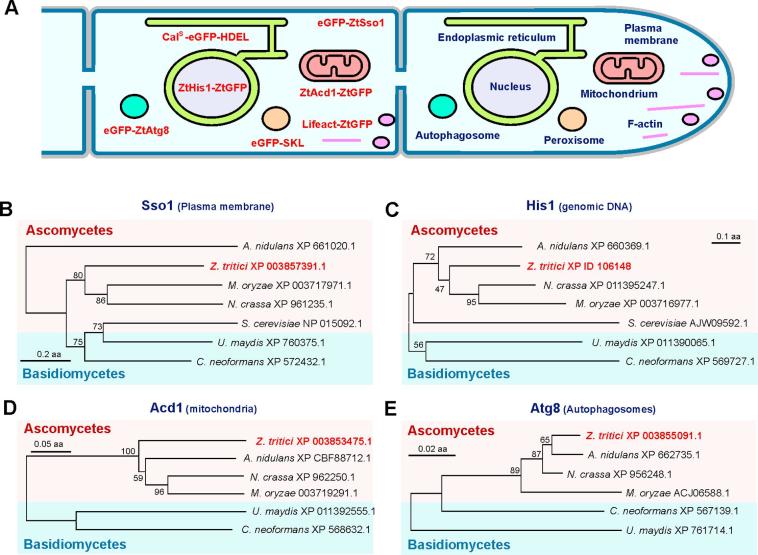
Markers for the plasma membrane, endoplasmic reticulum, nucleus, filamentous actin, mitochondria, autophagosomes and peroxisomes in *Z. tritici*. (A) Diagram depicting the localization of marker proteins established in this study. A fusion of eGFP and a syntaxin 2 homologue labels the plasma membrane (eGFP-ZtSso1). The lumen of the endoplasmic reticulum is labelled by a specific reporter that consists of a rabbit calreticulin signal peptide, fused to eGFP and the C-terminal ER-retention signal peptide HDEL (Cal^S^-eGFP-HDEL). A fusion of a histone H1 homologue and codon-optimised ZtGFP binds to genomic DNA in the nucleus (ZtHis1-ZtGFP). The LifeAct 17-amino acid peptide, derived from an *S. cerevisiae* actin-binding protein, was fused to codon-optimised ZtGFP and used to visualize filamentous actin (Lifeact-ZtGFP). Mitochondria were labelled by a short-branched acetyl-CoA dehydrogenase, fused to codon-optimised ZtGFP (ZtAcd1-ZtGFP). Autophagosomes were visualised by fusion protein of eGFP and a homologue of the autophagosome maturation protein Atg8 (eGFP-ZtAtg8). Finally, a reporter was targeted to peroxisomes, consisting of the peroxisome-targeting sequence 1, consisting of serine, lysine and leucine (SKL; (Gould et al., 1989), fused to the carboxy-terminus of eGFP (eGFP-SKL). (B) Phylogenetic trees comparing the predicted amino acid sequence of fungal homologues of the syntaxin ZtSso1, nucleus-associated histone H1 ZtHis1, puative acetyl-CoA dehydrogenase ZtAcd1 and the autophagosome-associated ZtAtg8. NCBI accession numbers are given behind species names (http://www.ncbi.nlm.nih.gov/pubmed). Maximum-likelihood trees were generated using MEGA5.2 (Tamura et al., 2011). Bootstrap values from 1000 rounds of calculation are indicated at branching points. Ascomycete protein sequences are highlighted by red background, basidiomycete protein sequences are highlighted by blue background. Bars indicate amino acid differences. (For interpretation of the references to colour in this figure legend, the reader is referred to the web version of this article.)

In this study we aimed to extend the selection of markers for labelling various organelles in *Z. tritici* (Fig.1A). Firstly, we identified *Z. tritici* homologues that were shown to localize specifically to plasma membrane, nuclear DNA, mitochondria and autophogosomes. We thus identified a homologue of the yeast plasma membrane syntaxin Sso1 (Aalto et al., 1993) that localizes in the fungal plasma membrane (Treitschke et al., 2010, Valdez-Taubas and Pelham, 2003). We identified ZtSso1 in the published genome of *Z. tritici* (protein ID 66031; NCBI accession number: XP_003857391.1), with 26.9% identity to yeast Sso1 and 28.1% identity to Sso1 from *U. maydis*. Similar to these proteins, ZtSso1 carries a syntaxin domain (*P* = 3.1e−20) and a SNARE domain (*P* = 1.2e−06; Table 1), and in a phylogenetic tree localizes within the group of other Sso1-homologues from ascomycete fungi (Fig.1B). To label nuclei, we identified a homologue of the histone H1 from *Neurospora crassa*, which binds to genomic DNA in this fungus (Freitag et al., 2004). The genome of *Z. tritici* encodes a histone H1 homologue (ZtHis1, protein ID: 106148), which groups with other ascomycete histone H1 proteins (Fig.1C) and that shares a linker H1 H5 domain (*P* = 3e−26) and 53.2% identity with histone H1 from *N. crassa* (Table 1). The putative mitochondrial acetyl-CoA dehydrogenase, ACAD, was identified in an extensive bioinformatic approach in *U. maydis* and was shown to localize in mitochondria of this fungus (Camoes et al., 2015). We used the predicted amino acid sequence of ACAD and identified a homologue in *Z. tritici*, which we named ZtAcd1 (Protein ID 104112; XP_003853475.1). The protein aligns closely with other ascomycete homologues (Fig.1D) and shares high sequence identity (54.0%), and three acyl-CoA dehydrogenase-typical domains (Acyl-CoA-dh_N, *P* = 1.6e−28; Acyl-CoA-dh_M, *P* = 4.6e−24; Acyl-CoA-dh_1, *P* = 8.4e−40) with *U. maydis* (Table 1). Finally, we made use of the previous identification of an Atg8 homologue in *Z. tritici*. This protein, named ZtAtg8 (protein ID 108219; NCBI accession number: XP_003855091.1), was initially identified in a two-hybrid approach as an interactor of the autophagy-related cysteine protease ZtAtg4 (Ma et al., 2015). We found that ZtAtg8 was incorrectly annotated, as sequence comparison with the Atg8 homologue in *M. oryzae* (Liu et al., 2010), revealed that the N-terminal 432 amino acids were not part of the open reading frame. The correct ZtAtg8 amino acid sequence is closely-related to other ascomycete Atg8-like proteins (Fig.1E) and shares 89.5% sequence identity and an Atg8 ubiquitin like domain (*P* = 3.0e−51) with Atg8 from *M. oryzae* (Table 1).

**Table 1:** Bioinformatics of marker proteins used in this study.

	Length^a^		Domains^b^		Identity^c^ (%)	Reference^d^
Sso1	*Z. tritici*	*U. maydis*	*Z. tritici*	*U. maydis*	28.1	Treitschke et al. (2010)
	340	418	Syntaxin (3.1e−20)	Syntaxin (3.3e−33)		
			SNARE (1.2e−06)	SNARE (9.1e−10)		
			Transmembrane	Transmembrane		
His1	*Z. tritici*	*N. crassa*	*Z. tritici*	*N. crassa*	53.2	Freitag et al. (2004)
	228	236	Linker H1 H5 (3e−26)	Linker H1 H5 (9e−27)		
Acd1	*Z. tritici*	*U. maydis*	*Z. tritici*	*U. maydis*	54.0	Camoes et al. (2015)
	443	465	Acyl-CoA_dh_N (1.6e−28)	Acyl-CoA_dh_N (1.1e−26)		
			Acyl-CoA_dh_M (4.6e−24)	Acyl-CoA_dh_M (1.5e−22)		
			Acyl-CoA_dh_1 (8.4e−40)	Acyl-CoA_dh_1 (2.6e−44)		
Atg8	*Z. tritici*	*M. oryzae*	*Z. tritici*	*M. oryzae*		
	119	123	Atg8 (3e−51)	Atg8 (5.4e−50)	89.4	Kershaw and Talbot (2009)

a Given in amino acids.

b Determined in PFAM (http://pfam.xfam.org/search/sequence) with error probability in brackets; transmembrane domain predicted in SMART (http://smart.embl-heidelberg.de/).

c Determined in EMBOSS Needle (http://www.ebi.ac.uk/Tools/psa/emboss_needle/).

d Reference reporting the use reference protein in other fungi.

### 2.2 Reporters for peroxisomes, endoplasmic reticulum and F-actin

We also aimed to visualize peroxisomes, endoplasmic reticulum (ER) and filamentous actin (F-actin). To this end, we designed reporter proteins that have been used successfully to visualize these organelle/filaments in other fungi. We fused the lifeact 17 amino acids peptide (MGVADLIKKFESISKEE), derived from the actin-binding protein ABP140p in *S. cerevisiae* (Riedl et al., 2008), to the codon-optimised ZtGFP, which was shown to be brighter and more photo-stable than eGFP (Kilaru et al., 2015c). Lifeact-GFP constructs have low toxicity and were previously used to visualize in filamentous fungi (Berepiki et al., 2010, Schuster et al., 2012, Wang and Shaw, 2016). To visualize peroxisomes, we fused a peroxisomal import signal SKL to eGFP. This PTS1-signal peptide is sufficient to target proteins to peroxisomes and has been used to visualize these organelles in fungi (Bartoszewska et al., 2011, Guimaraes et al., 2015, Salogiannis et al., 2016). Finally, we exploited a previously designed fluorescent reporter for the lumen of the ER. This marker consists of a Cal^s^ amino acid signal peptide of calreticulin from rabbit, fused to eGFP and a retention signal (HDEL, Pelham, 1990) that ensures residence the ER. This construct was used to visualize the ER network in *U. maydis* (Wedlich-Söldner et al., 2002).

### 2.3 Vectors for targeted integration of marker protein genes into the succinate dehydrogenase locus

**Fig. 2:**
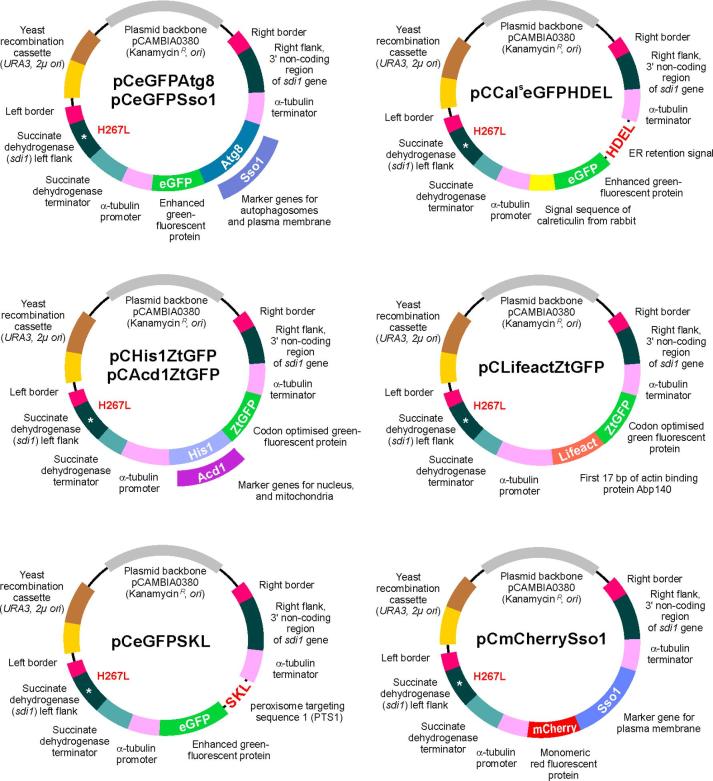
Vectors for targeted integration of fluorescent organelle marker genes into the succinate dehydrogenase locus of *Z. tritici*. The various vectors were designed by yeast recombination-based cloning (Kilaru and Steinberg, 2015) and are suitable for *Agrobacterium tumefaciens*-based transformation into *Z. tritici* (Zwiers and De Waard, 2001). During integration, the vectors are cleaved at the left and right border (indicated). Homologous recombination between the left flank of the succinate dehydrogenase gene, carrying the point mutation H267L, and the right flank of the succinate hydrogenase gene integrates the construct into the defined succinate dehydrogenase locus. The introduced H267L point mutation carboxin resistance and allows carboxin-based selection for correctly integrated constructs (Kilaru et al., 2015a). After integration, marker proteins are expressed under the promoter of the *Z. tritici* alpha-tubulin gene *zttub2* (Schuster et al., 2015b) and terminated by the *zttub2* terminator. Note that fragments are not drawn to scale.

Next, we generated vectors for integration of all 7 cellular marker genes into the genome of *Z. tritici* (Fig. 2). Random ectopic integration of the vectors bears the risk of uncontrolled and unrecognised gene disruption. We therefore designed vectors that allow controlled integration into the *Z. tritici* succinate dehydrogenase locus (Kilaru et al., 2015a). All vectors allow the expression of marker protein under the control of the constitutive *Z. tritici* α-tubulin (*tub2*, Schuster et al., 2015b) promoter (Fig. 2). The vectors were derived from the *Agrobacterium* binary vector pCAMBIA0380 (CAMBIA, Canberra, Australia), which enables *Agrobacterium tumefaciens*-based transformation into *Z. tritici*. This involves the 25 bp repeat sequences of the T-DNA borders (right and left border; Fig. 2). All plasmids carry the kanamycin resistance gene and an origin of replication for amplification in *E. coli* and *A. tumefaciens*. Targeted integration into the genomic succinate dehydrogenase *sdi1* locus was made possible by introducing a downstream *sdi1* sequence and a stretch of 3-prime non-coding sequence, downstream of *sdi1* (Fig. 2; right flank of *sdi1*). Importantly, the left *sdi1* flank contains a point mutation, which confers carboxin resistance and thus allows selection for successful integration (Fig. 2, H267L; Kilaru et al., 2015a). After homologous recombination, the mutation is incorporated into the complete *sdi1* gene. This method provides single integration of the construct, without the risk of disruption of other genes. Finally, all vectors contain a “yeast recombination cassette”, consisting of URA3 and 2µ *ori*, which is a consequence of yeast recombination-based cloning (Kilaru and Steinberg, 2015), and of no functional importance for integration into *Z. tritici*.

### 2.4 Vectors for random ectopic integration of marker protein genes into the genome of *Z. tritici*

**Fig. 3:**
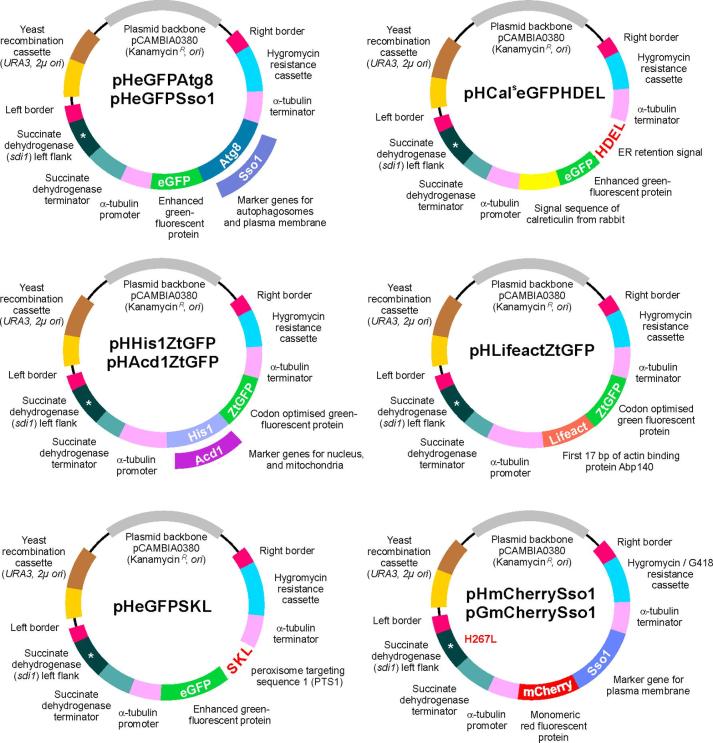
Vectors for random integration of fluorescent organelle marker genes into the genome of *Z. tritici*. The various vectors were designed by yeast recombination-based cloning (Kilaru and Steinberg, 2015) and are suitable for *Agrobacterium tumefaciens*-based transformation into *Z. tritici* (Zwiers and De Waard, 2001). During integration, the vectors are cleaved at the left and right border (indicated). The vectors carry the hygromycin resistance cassette and randomly integrate into the genome. Note that these vectors were derived from carboxin resistance conferring vectors (Fig. 2). As such they contain the left flank of the succinate dehydrogenase gene, carrying the mutation H267L and the succinate dehydrogenase terminator. However, these fragments are of no significance.

Next, we generated a set of vectors that carry the DNA encoding the fluorescent marker proteins, but instead of carboxin carry a hygromycin resistance-conferring cassette. These plasmids lack the 3′ non-coding region of the *sdi1* gene (Fig. 3), which prevents them from integrating into the *sdi1* locus. Instead, they randomly integrate into the genome, which allows transformation into wild-type *Z. tritici*, but also into strains that already carry another resistance cassette, such as *nptII* (neomycin phosphotransferase; Geneticin (=G418) resistant) or *bar* (phosphinothricin acetyltransferase; Basta resistant) or *sdi1^R^* (mutated allele of succinate dehydrogenase, H267, see above). All vectors still contain the *sdi1* downstream sequence (*sdi1* left flank and terminator, Fig. 3), which is reminiscent from the original vector set and has no functional significance.

ZtSso1 is a useful marker for defining individual cells in the multi-cellular macropycnidiospores. For co-localization studies, with the GFP-carrying markers, we generated vectors that contain ZtSso1 fused to mCherry. This red-fluorescent protein was shown to be the best-suited for studies in *Z. tritici* (Schuster et al., 2015a). We constructed vector pHmCherrySso1, for random integration and hygromycin-based selection, pGmCherry Sso1 for random integration into the genome and Geneticin (=G418)-based selection (Fig. 3), and pCmCherrySso1, carrying for targeted integration into the succinate dehydrogenase locus and selection by carboxin (Fig. 2, lower right).

### 2.5 Visualization of fluorescently labelled organelles and F-actin in *Z. tritici*

**Fig. 4:**
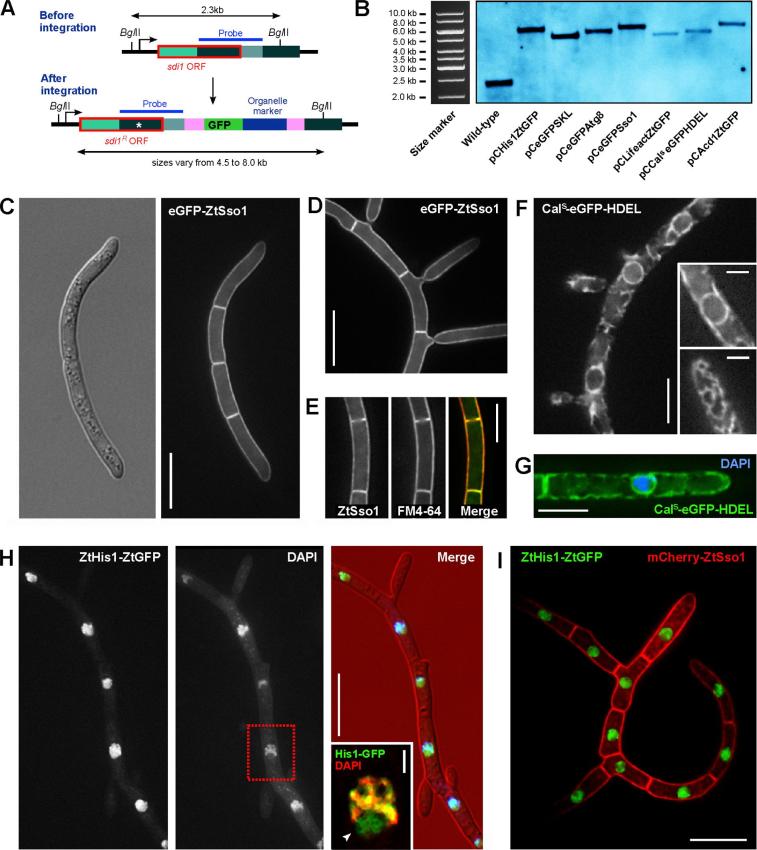
Localization of markers for the plasma membrane, the endoplasmic reticulum and the nucleus in *Z. tritici*. (A) Diagram showing the genomic organisation of the succinate dehydrogenase locus before and after integration of marker plasmid. After digestion with the restriction enzyme *Bgl*II, the succinate dehydrogenase locus can be recognised in Southern blots as a 2.3 kilo base DNA fragment (see region of probing indicated by a blue bar and “Probe”). After homologous integration, the point-mutated C-terminal half the succinate dehydrogenase gene is integrated into the *sdi1* locus, which results in a carboxin-resistant allele of the succinate dehydrogenase. After digestion with *Bgl*II, fragments of 4.5–8.0 kB are generated. (B) Southern blot showing integration of all plasmids, shown in Fig. 2, into the succinate dehydrogenase locus. Single bands of 4.5–8.0 kB bases are visible, confirming that one copy was integrated into the defined locus. (C) *Z. tritici* cells that express the plasma membrane marker eGFP-ZtSso1. The marker localizes in the peripheral plasma membrane and defines the outer limits of the multi-cellular cell structure. Bar represents 10 µm. (D) *Z. tritici* cells that form lateral “buds” and express the plasma membrane marker eGFP-ZtSso1. The marker shows the “bud necks” at the base of newly formed cells. Bar represents 10 µm. (E) Brief staining of eGFP-ZtSso1 expressing cells the lipophilic dye FM4-64. Merging of the green and red fluorescence revealed perfect co-localization, confirming the marker locates exclusively to the plasma membrane. Bar represents 5 µm. (F) *Z. tritici* cells expressing the endoplasmic reticulum marker Cal^s^-eGFP-HDEL. The fluorescent protein is labelling a peripheral network (lower inset) and a spherical structure in the middle of each cell (upper inset). Bars represent 5 µm (main images) and 2 µm (insets). (G) DAPI-stained *Z. tritici* cell that expresses the endoplasmic reticulum marker Cal^s^-eGFP-HDEL. The dye-stained genomic DNA is surrounded by the nuclear envelope, which is part of the endoplasmic reticulum, labelled by Cal^s^-eGFP-HDEL. Bar represents 5 µm. (H) *Z. tritici* cells that express the nuclear DNA-binding marker His1-ZtGFP. The marker localizes in nuclei, which are counterstained by DAPI. Merging both fluorescent channels reveals that the fluorescent histone H1 is also located in the nucleolus, which does not contain chromosomal DNA and thus is not stained by DAPI (inset). Bars represent 10 µm (main images) and 1 µm (inset). (G) Co-localization of the plasma membrane, labelled with the red-fluorescent marker mCherry-ZtSso1, and the nuclear marker protein ZtHis1-ZtGFP. Bar represents 10 µm.

**Supplementary Fig. S1:**
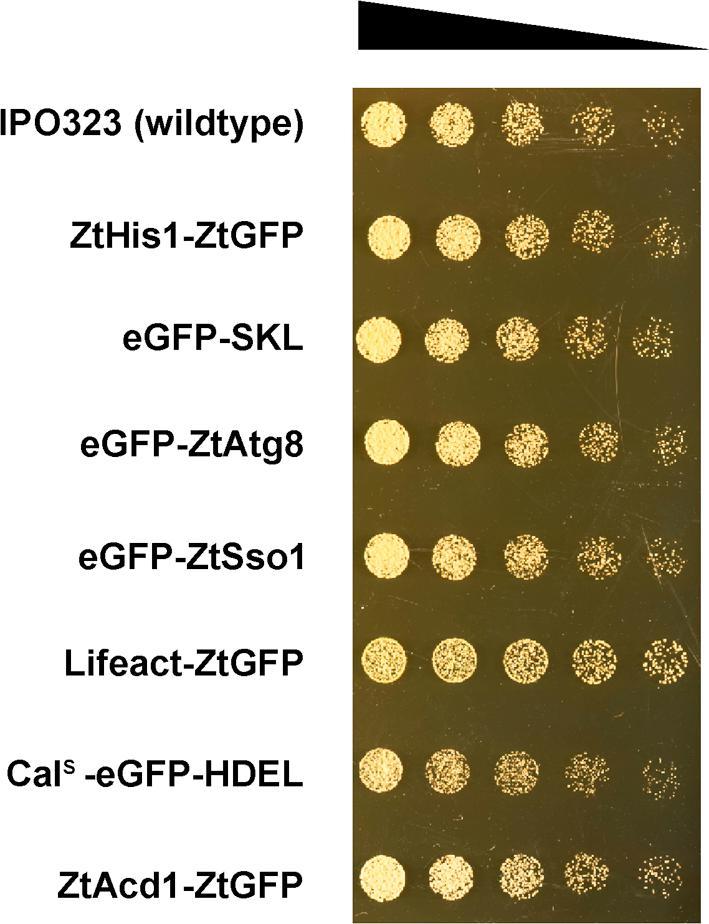


To visualize the plasma membrane, the ER, nuclei, F-actin, mitochondria, peroxisomes and autophagosomes, we transformed the vectors pCeGFPSso1, pCCal^s^eGFPHDEL, pCHis1ZtGFP, pCLifeactZtGFP, pCAcd1ZtGFP, pCeGFPSKL and pCeGFPAtg8 into *Z. tritici* wildtype strain IPO323 using *A. tumefaciens*-mediated transformation (Zwiers and De Waard, 2001). Homologous integration of these vectors into succinate dehydrogenase introduced a carboxin-resistance conferring mutation into the endogenous *sdi1* gene (Fig.4A). Positive integration of the entire construct into the *sdi1* locus was confirmed by Southern blot analysis, where *Bgl*II-digested genomic DNA fragments shifted from ∼2.5 to 4.5–8.0 kilo bases (Fig.4A and B). Integration of all vectors into the wildtype strain IPO323 did not have an obvious effect on plate growth (Supplementary Fig. S1), although integration of pCLifeactZtGFP resulted in a tendency to melanise (not shown).

Next, we analysed the cellular localization of all fluorescent markers by epi-fluorescent microscopy. The marker eGFP-ZtSso1 labelled the edge of the cell and septa (Fig.4C). The even distribution at the cell periphery is in agreement with its proposed localization in the plasma membrane in *Aspergillus nidulans* (Taheri-Talesh et al., 2008). Using eGFP-ZtSso1 confirmed previous observations that *Z. tritici* macropycnidiospores are multi-cellular (Steinberg, 2015) and grow by lateral “budding”, a process named “microcycle conidiation” (Jung et al., 2014; Fig.4D). The localization in the plasma membrane was further confirmed by co-visualization of eGFP-ZtSso1 and the lipophilic probe FM4-64 (Fig.4E), which enters the fungal plasma membrane before uptake into the cell (Fischer-Parton et al., 2000, Wedlich-Söldner et al., 2000).

The marker Cal^s^-eGFP-HDEL is a synthetic reporter for the lumen of the ER, consisting of a 17 amino acid ER signal peptide from rabbit calreticulin (Fliegel et al., 1989), fused to eGFP and an ER-retention signal (HDEL, (Pelham, 1990). This reporter was used previously in the basidiomycete *U. maydis* to investigate the dynamic behaviour of ER tubules and the involvement of molecular motors in formation of the peripheral network (Wedlich-Söldner et al., 2002). When the same construct was expressed in IPO323, it labelled a peripheral network and a spherical structure in the cell (Fig.4F, insets show optical section in the cell middle and upper cell periphery, respectively). This localization is in full agreement with the organisation of the ER in *U. maydis* (Wedlich-Söldner et al., 2002). It was suggested that the spherical ER structure represents the nuclear envelope in *U. maydis* (Wedlich-Söldner et al., 2002). Indeed, in IPO323, the spherical ER surrounds genomic DNA, stained with the dye 4′,6-diamidino-2-phenylindole (DAPI; Fig.4G). Thus, Cal^s^-eGFP-HDEL is a fluorescent marker of the ER in *Z. tritici*.

Histones are genomic DNA-associated proteins that have conserved roles in genomic DNA folding and transcription regulation (Grunstein, 1990). In *N. crassa*, a type 1 histone was used to visualize the nuclear DNA (Freitag et al., 2004). We identified and expressed a close homologue of histone H1 in *Z. tritici* (ZtHis1). Consistent with the expected role in chromosomal DNA organisation and transcription, the fluorescent marker ZtHis1-ZtGFP labels the nucleus and co-localizes with the DNA-specific dye DAPI (Fig.4H). In addition, the marker is found in the nucleolus, which is identified by the exclusion of DAPI staining (Fig.4H, arrowhead in inset; overlay image was highly deconvolved to reduce background fluorescence). Such localization was reported for histone H1 in *Drosophila* fruit flies, and is indicative of a role in rDNA transcription during ribosome formation (Tani et al., 2016). Next, we co-expressed ZtHis1-ZtGFP and a red-fluorescent mCherry-ZtSso1 (vector pGmCherrySso1, Fig. 3). Random integration of pGmCherrySso1 allowed co-visualization of nuclei and the plasma membrane, which demonstrates that each cell compartment in a multi-cellular macropycnidiospore contains a single nucleus (Fig.4I).

**Fig. 5:**
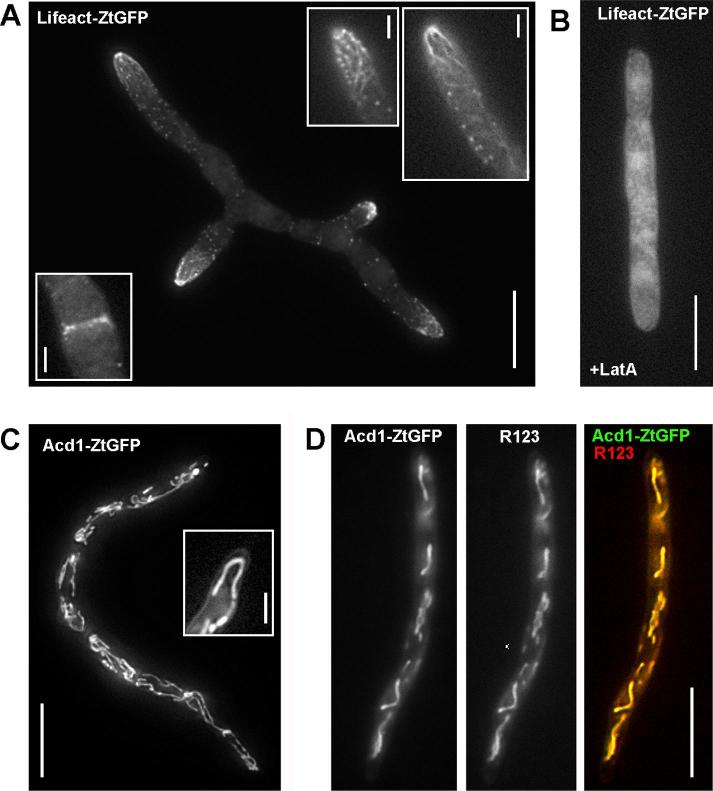
Localization of markers for actin filaments and mitochondria. (A) *Z. tritici* cells that express a fusion of the F-actin-specific Lifeact peptide (MGVADLIKKFESISKEE) and codon-optimised ZtGFP (Lifeact-ZtGFP). The F-actin probe labels patches and cables (upper right inserts), as well as the contractile ring at growing septa (lower left insert). This localization is characteristic for F-actin in filamentous fungi (Berepiki et al., 2011). Bars represent 10 µm (main image), and 2 µm (all insets). See Supplementary Video 1 and 2. (B) *Z. tritici* cells that express Lifeact-ZtGFP after 45 min treatment with 10 µM latrunculin A. The drug depolymerises F-actin and results in unspecific cytoplasmic background fluorescence, confirming that Lifeact-ZtGFP labels F-actin in *Z. tritici*. Bar represents 10 µm. (C) *Z. tritici* cells that express a fusion of the putative mitochondrial acetyl-CoA dehydrogenase ZtAcd1 and codon-optimised ZtGFP (ZtAcd1-ZtGFP). The marker locates to elongate mitochondria. Image is a maximum-projection of a Z-axis image stack and was 2D-deconvolved. Bars represent 10 µm (main image), and 3 µm (inset). (D) Staining of ZtAcd1-ZtGFP expressing cells with the red-fluorescent mitochondrial marker dye Rhodamine 123. The co-localization after merging both fluorescent channels confirms that ZtAcd1-ZtGFP resides in mitochondria. Bar represents 10 µm.

The F-actin cytoskeleton of fungi consists of actin patches, actin cables and contractile rings at sites of septation (Berepiki et al., 2011, Heath, 1994). We used the reporter Lifeact-ZtGFP, which consists of the F-actin-binding Lifeact peptide, derived from the *S. cerevisiae* actin-binding protein ABP140p (Riedl et al., 2008), and a ZtGFP, codon-optimised for use in *Z. tritici* (Kilaru et al., 2015c). When expressed in IPO323, the fluorescent marker labelled fine filaments at the growing cell end and at the site of septum formation (Fig.5A; Video 1, 2). In addition, the Lifeact-ZtGFP reporter stained actin patches, which indicate sites of formation of endocytic transport vesicles at the plasma membrane. These localizations were disrupted when cells were treated with the F-actin inhibitor Latrunculin A (Fig.5B), which disassemble F-actin in fungi (Ayscough et al., 1997, Fuchs et al., 2005). Thus, Lifeact-ZtGFP is a reliable marker for visualization of F-actin in living cells of *Z. tritici*.

β-Oxidation of fatty acid esters is an important function of mitochondria in animal cells (Schulz, 1991). It relies on acyl-CoA dehydrogenases (ACADs), which have important roles in inter-molecular electron transfer for ATP production in the respiratory chain. Recently, a mitochondrial short-branched ACAD was identified in mitochondria of the fungus *U. maydis* (Camoes et al., 2015). We used the predicted amino acid sequence of this ACAD to identify a highly homologous putative acyl-CoA dehydrogenase in the genome of *Z. tritici*. A fusion of this putative enzyme, named Acd1, to codon-optimised ZtGFP localized to elongated structures that were evenly scattered in the cytoplasm of IPO323 cells (Fig.5D). This shape and distribution is reminiscent of fungal mitochondria (Steinberg and Schliwa, 1993, Steinberg and Schuster, 2011). We confirmed the mitochondrial localization of ZtAcd1-ZtGFP by co-staining with the mitochondria-specific dye Rhodamine 123 that targets the mitochondrial membrane potential (Siemens et al., 1982; Fig.5D). Thus, ZtAcd1-ZtGFP is a reliable marker for mitochondria. It is worth noting that the localization of a putative ACAD in mitochondria of the ascomycete *Z. tritici* indicates that mitochondrial β-oxidation is a general feature of fungi, which is in contrast to previous conclusions that fatty acid ester degradation is restricted to fungal peroxisomes (Poirier et al., 2006).

**Fig. 6:**
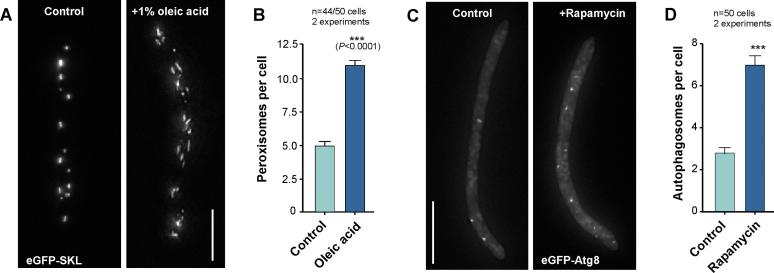
Localization of markers for peroxisomes and autophagosomes. (A) eGFP-SKL expressing *Z. tritici* cells in control cells and cells that were exposed to 1% (v/v) oleic acid, which induces peroxisome formation in fungi (Camoes et al., 2015, Goodman et al., 1990). The number eGFP-SKL signals increase and their shape changes upon oleic acid treatment, suggesting that the marker is, indeed, located in peroxisomes. Bar represents 10 µm. (B) Graph showing numbers of eGFP-SKL signals in control cells and cells treated with 1% (v/v) oleic acid for 3 h. The number of signals significantly increases (Student’s *t*-test; error probability *P* < 0.0001). Mean ± standard error of the mean is depicted, sample size is indicated. (C) *Z. tritici* cells that express eGFP fused to the autophagosomal protein ZtAtg8. In 47-treated control cells, only few eGFP-ZtAtg8 signals are visible. Treatment with the mTOR inhibitor rapamycin, which was reported to induce autophagy (Dumont and Su, 1996), increases the number of fluorescent signals. This confirms that eGFP-Atg8 locates to autophagosomes. Images are maximum-projections of Z-axis image stacks and were 2D-deconvolved; bar represents 10 µm. (D) Graph showing numbers of eGFP-Atg8 labelled autophagosomes in control cells and cells treated for 4 h with 200 nM rapamycin. The number of signals significantly increases (Student’s *t*-test; *** = error probability *P* < 0.0001). Mean ± standard error of the mean is depicted, sample size is indicated.

Peroxisomes are essential organelles that perform a broad range of functions in fungi. This includes fatty acid β-oxidation and hydrogen peroxide metabolism, synthesis of secondary metabolites, including antibiotics, biotin and toxins (van der Klei and Veenhuis, 2013). Proteins are targeted into peroxisomes by a COOH-terminal tripeptide peroxisomal targeting signals (Subramani, 1992). The common PTS1 targeting signal consists of serine, lysine and leucine (SKL). When fused to the carboxy-terminus of GFP this peptide targets the fusion protein into fungal peroxisomes (Guimaraes et al., 2015). We expressed eGFP-SKL in *Z. tritici* wildtype strain IPO323. The fluorescent marker concentrated in evenly-distributed vesicular structures (Fig.6A). This distribution was described for peroxisomes in *A. nidulans* (Salogiannis et al., 2016) and *U. maydis* (Guimaraes et al., 2015, Lin et al., 2016). In fungi, peroxisome formation is induced by treatment with oleic acid (Camoes et al., 2015, Goodman et al., 1990). In agreement, the number of eGFP-SKL-positive signals significantly increased after 3 h treatment with 1% (v/v) oleic acid (Fig.6A and B). We conclude that the eGFP-SKL reporter is targeted to peroxisomes.

Finally, we visualised autophagosomes in *Z. tritici*. Autophagy is a starvation-induced pathway that delivers bulk cytoplasm and organelles, such as peroxisomes, to the vacuole for degradation and recycling (Huang and Klionsky, 2002). In the rice blast fungus *Magnaporthe oryzae*, autophagy is essential for plant infection and it involves the autophagosome maturation protein Atg8 (Kershaw and Talbot, 2009). A fluorescent GFP-MoAtg8 fusion protein labels punctate autophagosomes in this fungus (Kershaw and Talbot, 2009). GFP-Atg8 was also used to study autophagosome biogenesis in *A. nidulans* (Pinar et al., 2013). Expression of eGFP-ZtAtg8 in IPO323 resulted in the appearance of small fluorescent vesicles (Fig.6C). To confirm that these structures are indeed autophagosomes, we induced autophagy in eGFP-ZtAtg8-expessing cells by treatment with rapamycin. This drug inhibits the mTOR kinase (Dumont and Su, 1996), which represses autophagy in the yeast *S. cerevisiae* (Noda and Ohsumi, 1998) and appressorium formation in *Magnaporthe oryzae* (Marroquin-Guzman and Wilson, 2015). Treatment with 200 nM rapamycin for 4 h was shown to increase the number of Atg8-positive autophagosomes in *Aspergillus oryzae* (Kikuma et al., 2006). When the same conditions were applied, *Z. tritici* cells showed a significant increase in the number of eGFP-ZtAtg8 signals in the cells (Fig.6D), which confirms that the marker labels autophagosomes in *Z. tritici*.

## 3 Conclusion

In this report, we provide 8 additional fluorescent markers for live cell imaging of the plant pathogen *Z. tritici*. This significantly extends our collection of molecular tools for studying this important cause of Septoria tritici blotch in wheat (Table 3). In recent years, live cell imaging of fungal cellular organisation and protein dynamics has significantly increased our understanding of infection strategies in fungal pathogens, such as the rice blast fungus (Dagdas et al., 2012, Giraldo et al., 2013, Gupta et al., 2015, Kankanala et al., 2007) and the corn smut fungus (Bielska et al., 2014, Djamei et al., 2011, Hemetsberger et al., 2012). *Z. tritici* infections pose even more serious threats to food security in Europe (Fones and Gurr, 2015, Torriani et al., 2015). However, despite its high economic importance, almost nothing is known about the cell biology of this fungus (Steinberg, 2015). The molecular markers presented in this report are, therefore, valuable tools to foster a better understanding of *Z. tritici* and its infection strategies.

## 4 Methods

### 4.1 Bacterial and fungal strains and growth conditions

*Escherichia coli* strain DH5α was used for the maintenance of plasmids.

*A. tumefaciens* strain EHA105 (Hood et al., 1993) was used for maintenance of plasmids and subsequently for *A. tumefaciens*-mediated transformation of *Z. tritici*. *E. coli* and *A. tumefaciens* were grown in DYT media (tryptone, 16.0 g/l; yeast extract, 10 g/l; NaCl, 5 g/l; with 20 g/l agar added for preparing the plates) at 37 °C and 28 °C respectively. The fully sequenced *Z. tritici* wild-type isolate IPO323 (Goodwin et al., 2011, Kema and van Silfhout, 1997) was used as recipient strain for the genetic transformation experiments. The isolate was inoculated from stocks stored in NSY glycerol (nutrient broth, 8 g/l; yeast extract, 1 g/l; sucrose, 5 g/l; glycerol, 700 ml/l), at −80 °C onto solid YPD agar (yeast extract, 10 g/l; peptone, 20 g/l; glucose, 20 g/l; agar, 20 g/l) and grown at 18 °C for 4–5 days.

### 4.2 Identification of *Z. tritici* homologues and bioinformatics

To identify homologues of the chosen marker proteins, we screened the published sequence of *Z. tritici* (http://genome.jgi.doe.gov/Mycgr3/Mycgr3.home.html), using the provided BLASTP function and the *U. maydis* protein sequences of Acad (NCBI accession number: XP_011392555.1) Sso1 (NCBI accession number: XP_003857391.1), *M. oryzae* protein sequence of Atg8 (NCBI accession number: ACJ06588.1) and *N. crassa* protein sequence of H1 (NCBI accession number: XP_011395247.1). Sequences were obtained from the NCBI server (http://www.ncbi.nlm.nih.gov/pubmed) and comparison was done using CLUSTAL W (http://www.ebi.ac.uk/Tools/msa/clustalw2/) and EMBOSS Needle (http://www.ebi.ac.uk/Tools/psa/emboss_needle/) and domain structures were analysed in PFAM (http://pfam.xfam.org/search/sequence). Phylogenetic trees were generated in MEGA5.2, using a Maximum likelihood algorithm, followed by 1000 bootstrap cycles (http://www.megasoftware.net/; (Tamura et al., 2011).

### 4.3 Molecular cloning

All the vectors used in this study were generated by *in vivo* recombination in the yeast *S. cerevisiae* DS94 (MATα, *ura3-52*, *trp1-1*, *leu2-3*, *his3-111*, and *lys2-801* (Tang et al., 1996), following published procedures (Kilaru and Steinberg, 2015, Raymond et al., 1999). For all the recombination events, the fragments were amplified with 30 bp homologous sequences to the upstream and downstream of the fragments to be cloned (see Table 2 for primer details). PCR reactions and other molecular techniques followed standard protocols (Sambrook and Russell, 2001). All restriction enzymes and reagents were obtained from New England Biolabs Inc (NEB, Herts, UK).

**Table 2:** Primers used in this study.

Primer name	Direction	Sequence (5′ to 3′)^a^
SK-Sep-10	Sense	*TGGCAGGATATATTGTGGTGTAAACAAATT*GACCTTCCACATCTACCGATGG
SK-Sep-13	Antisense	CTTCCGTCGATTTCGAGACAGC
SK-Sep-14	Sense	*CATTTGCGGCTGTCTCGAAATCGACGGAAG*GCAGTCGACGCCAGATGATGG
SK-Sep-15	Antisense	*GGTGAACAGCTCCTCGCCCTTGCTCACCAT*GGCGATGGTGGTATGCGGATG
SK-Sep-16	Sense	*ATGGTGAGCAAGGGCGAGGAG*
SK-Sep-44	Antisense	*CCACAAGATCCTGTCCTCGTCCGTCGTCGCTTAAAGCTTCGACTTGTACAGCTC*
SK-Sep-46	Sense	GAAGTCTGCGGCAGCTCGCAC
SK-Sep-47	Antisense	GGCGATGGTGGTATGCGGATG
SK-Sep-67	Sense	*ATCACTCTCGGCATGGACGAGCTGTACAAG*ATGCGCTCCAAGTTCAAGGACG
SK-Sep-68	Antisense	*CCACAAGATCCTGTCCTCGTCCGTCGTCGC*CTATACGGCCTCGCCGAAGGT
SK-Sep-101	Sense	*CATCACTCACATCCGCATACCACCATCGCC*ATGGTCTCCAAGGGCGAGGAG
SK-Sep-102	Antisense	*CCACAAGATCCTGTCCTCGTCCGTCGTCGC*TTACTTGTAGAGCTCGTCCATGC
SK-Sep-128	Sense	C*TCTCATAAGAGCTTGGCTGTCGACTCCTC*GAATTCGAGCTCGGTACCCAACT
SK-Sep-129	Antisense	*CTTTTCTCTTAGGTTTACCCGCGTTGAAGT*GCGTTAACACTAGTCAGATCTACC
SK-Sep-160	Sense	*ATCACTCTCGGCATGGACGAGCTGTACAAG*ATGTCGAACTACAACCAGTACTCG
SK-Sep-161	Antisense	*CCACAAGATCCTGTCCTCGTCCGTCGTCGC*TCAATTCTTACTCGGCGGGCCC
SK-Sep-239	Antisense	*GTAGCCCGAGTACTGGTTGTAGTTCGACAT*CTTGTACAGCTCGTCCATGCCG
SK-Sep-287	Sense	*CATCACTCACATCCGCATACCACCATCGCC*ATGCCTCCCAAGAAAGTCACCAC
SK-Sep-288	Antisense	*GGTGAAGAGCTCCTCGCCCTTGGAGACCAT*TGCCTTCTTGGGAGTGGCGGC
SK-Sep-415	Sense	*CTCTCATAAGAGCTTGGCTGTCGACTCCTC*GCGGCTTCGAATCGTGGCTAC
SK-Sep-416	Antisense	*TAAACGCTCTTTTCTCTTAGGTTTACCCGC*ATCATCATGCAACATGCATGTACTG
SK-Sep-425	Sense	*CATCACTCACATCCGCATACCACCATCGCC*ATGTTGCTCCCTGTGCCGCTGC
SK-Sep-426	Antisense	*CCACAAGATCCTGTCCTCGTCCGTCGTCGC*TTACAGCTCGTCGTGCTTGTACAG
SK-Sep-427	Sense	*ctcctccttggagatggactcgaacttcttgatgaggtcggcgacgccca*tggcgatggtggtatgcggatg
SK-Sep-428	Antisense	*atgggcgtcgccgacctcatcaagaagttcgagtccatctccaaggaggaga*tggtctccaagggcgaggagc
SK-Sep-431	Sense	*CATCACTCACATCCGCATACCACCATCGCC*ATGGCATCTGTCACACGAACTCTT
SK-Sep-432	Antisense	*GGTGAAGAGCTCCTCGCCCTTGGAGACCAT*TGTCGTGTACTGCTTCTGCAACAG

a *Italics* indicates sequence, complementary with the DNA fragment, that is to be ligated by homologous recombination in *S. cerevisiae*.

**Table 3:** Fluorescent protein markers in *Z. tritici.*

Target	Marker	Reference
*1. Organelles*		
Nuclear DNA	His1-ZtGFP	This study
Peroxisomes	eGFP-SKL	This study
Mitochondria	Acd1-ZtGFP	This study
Vacuoles	eGFP-Rab7	Kilaru et al. (2015b)
Autophagosomes	eGFP-Atg8	This study
ER matrix	eGFP-HDEL	This study
Early endosome	eGFP-Rab5	Kilaru et al. (2015b)
Late endosomes	eGFP-Rab7	Kilaru et al. (2015b)
Recycling carriers	eGFP-Rab11	Guo et al. (2015)
Secretory vesicles	ZtGFP-Sec4	Guo et al. (2015)
Plasma membrane§	eGFP-Sso1, mCherry-Sso1	This study

*2. Polarity markers*		
Spitzenkörper	ZtGFP-Mlc1	Guo et al. (2015)
Polarisome	ZtGFP-Spa2	Guo et al. (2015)
Exocyst	ZtGFP-Exo70	Guo et al. (2015)

*3. Cytoskeleton*		
F-actin	Lifact-ZtGFP	This study
Actin patches	Fim1-eGFP	Kilaru et al. (2015b)
Microtubules§	eGFP-Tub2, mCherry-Tub2	Schuster et al. (2015b)
Microtubule plus-end	EB1-eGFP	Schuster et al. (2015b)
Spindle pole body	Grc1-eGFP	Schuster et al. (2015b)

*4. Miscellaneous*		
Cytoplasm§	eGFP, AcGFP, ZtGFP, mCherry, mRFP, TagRFP, tdTomato	Kilaru et al., 2015a, Kilaru et al., 2015c; Schuster et al. (2015a)

His1 = Histone 1; SKL = PTS1 signal peptide; Acd1 = acyl-CoA dehydrogenase; Rab5, Rab7, Rab11 = small endosomal GTPases; Atg8 = autophagosome maturation protein; HDEL = endoplasmic reticulum retention signal; Sec4 = small GTPase; Sso1 = syntaxin 1; Mlc1 = myosin light chain 1; Spa2 = Polarisome scaffold protein; Exo70 = Exocyst subunit; Lifeact = 17 residues from the actin binding protein ABP140p; Fim1 = fimbrin; Tub2 = αtubulin; EB1 = microtubule plus end binding protein; Grc1 = γtubulin ring complex protein; eGFP = enhanced green fluorescent protein from *Aequorea victoria*; AcGFP = green fluorescent protein from *Aequorea coerulescens*; ZtGFP = codon-optimised eGFP; mCherry, mRFP, TagRFP, tdTomato = red fluorescent proteins.

§ Fused to green and red fluorescent proteins.

### 4.4 Targeted ectopic integration vectors to visualize various organelles

The vectors pCeGFPAtg8 and pCeGFPSso1 contains *egfp* fused to the full-length *ztatg8* and *ztsso1* under the control of constitutive *zttub2* promoter and terminator sequences for targeted integration in to the *sdi1* locus of *Z. tritici* by using carboxin as selection agent. A 14,907 bp fragment of pCeGFPTub2 (Schuster et al., 2015b); digested with *Xho*I) and either 512 bp full-length *ztatg8* gene (amplified with SK-Sep-67 and SK-Sep-68; Table 2) or 1080 bp full-length *ztsso1* gene (amplified with SK-Sep-160 and SK-Sep-161; Table 2) were recombined in yeast *S. cerevisiae* to obtain the vectors pCeGFPAtg8 and pCeGFPSso1 respectively (Fig. 2).

The vector pCCal^s^eGFPHDEL contains *egfp* fused to the signal sequence of calreticulin from rabbit Cal^s^ and ER retention signal HDEL under the constitutive *zttub2* promoter and terminator sequences for targeted integration in to the *sdi1* locus of *Z. tritici* using carboxin as selection agent. A 12,530 bp fragment of pCeGFPTub2 (Schuster et al., 2015b); digested with *Bsr*GI), 1149 bp *Z. trirtici* α-tubulin promoter (amplified with SK-Sep-14 and SK-Sep-15; Table 2) and 51 bp Cal^s^, 717 bp *egfp* and 12 bp encoding HDEL (Cal^s^eGFPHDEL sequence; amplified with SK-Sep-425 and SK-Sep-426; Table 2) were recombined in yeast *S. cerevisiae* to obtain the vector pCCal^s^eGFPHDEL (Fig. 2).

The vectors pCHis1ZtGFP and pCAcd1ZtGFP contains *ztgfp* fused to the full-length *zthis1* and *ztacd1* under the control of constitutive *zttub2* promoter and terminator sequences for targeted integration in to the *sdi1* locus of *Z. tritici* by using carboxin as selection agent. A 12,971 bp fragment of pCZtGFP (Kilaru et al., 2015c); digested with *Zra*I), 977 bp *zttub2* promoter (amplified with SK-Sep-46 and SK-Sep-47; Table 2), 720 bp *ztgfp* (amplified with SK-Sep-101 and SK-Sep-102; Table 2) and either 1235 bp full-length *zthis1* gene (amplified with SK-Sep-287 and SK-Sep-288; Table 2) or 1382 bp full-length *ztacd1* gene (amplified with SK-Sep-431 and SK-Sep-432; Table 2) or were recombined in yeast *S. cerevisiae* to obtain the vectors pCHis1ZtGFP and pCAcd1ZtGFP respectively (Fig. 2).

The vector pCLifeactZtGFP contains *ztgfp* fused to the first 17 amino acids of the actin-binding region of ABP140p in *S. cerevisiae* (MGVADLIKKFESISKEE; Riedl et al., 2008) under the control of constitutive *zttub2* promoter and terminator sequences for targeted integration in to the *sdi1* locus of *Z. tritici* by using carboxin as selection agent. Chimeric primers SK-Sep-427 and SK-Sep-428 contain 17 amino acid lifeact sequences with *Z. tritici*-codon optimised nucleotides (ATG GGC GTC GCC GAC CTC ATC AAG AAG TTC GAG TCC ATC TCC AAG GAG GAG). A 12,971 bp fragment of pCZtGFP (Kilaru et al., 2015a, Kilaru et al., 2015b, Kilaru et al., 2015c; digested with *Zra*I), 977 bp *zttub2* promoter (amplified with SK-Sep-46 and SK-Sep-427; Table 2) and the 727 bp *ztgfp* gene (amplified with SK-Sep-428 and SK-Sep-102; Table 2) were recombined in yeast *S. cerevisiae* to obtain the vectors pCLifeactZtGFP (Fig. 2).

The vector pCeGFPSKL contains *egfp* fused to the tripeptide SKL that serve as peroxisome targeting signal under the constitutive *zttub2* promoter and terminator sequences for targeted integration in to the *sdi1* locus of *Z. tritici* using carboxin as selection agent. A 12,530 bp fragment of pCeGFPTub2 (Schuster et al., 2015b); digested with *Bsr*GI), 1149 bp *Z. trirtici* α-tubulin promoter (amplified with SK-Sep-14 and SK-Sep-15; Table 2) and 717 bp *egfp* and 9 bp encoding SKL (peroxisome targeting signal sequence; amplified with SK-Sep-16 and SK-Sep-44; Table 2) were recombined in yeast *S. cerevisiae* to obtain the vector pCeGFPSKL (Fig. 2).

The vector pCmCherrySso1 contains *mCherry* fused to the full-length *ztsso1* under the control of constitutive *zttub2* promoter and terminator sequences for targeted integration in to the *sdi1* locus of *Z. tritici* by using carboxin as selection agent. A 13,457 bp fragment of pCeGFPSso1 (Fig.2A; digested with *Bsr*GI), 977 bp *zttub2* promoter (amplified with SK-Sep-46 and SK-Sep-47; Table 2) and 717 bp mCherry (amplified with SK-Sep-101 and SK-Sep-239; Table 2) were recombined in yeast *S. cerevisiae* to obtain the vector pCmCherrySso1 (Fig. 2).

### 4.5 Random ectopic integration vectors to visualize various organelles

The vectors pHeGFPAtg8 and pHeGFPSso1 (Fig. 3) contains *egfp* fused to the full-length *ztatg8* and *ztsso1* under the control of constitutive *zttub2* promoter and terminator sequences for random ectopic integration into the genome of *Z. tritici* by using hygromycin B as selection agent. The vector pHCal^s^eGFPHDEL (Fig. 3) contains *egfp* fused to the signal sequence of calreticulin from rabbit Cal^s^ and ER retention signal HDEL under the constitutive *zttub2* promoter and terminator sequences for random ectopic integration into the genome of *Z. tritici* by using hygromycin B as selection agent. The vectors pHHis1ZtGFP and pHAcd1ZtGFP (Fig. 3) contains *ztgfp* fused to the full-length *zthis1* and *ztacd1* under the control of constitutive *zttub2* promoter and terminator sequences for random ectopic integration into the genome of *Z. tritici* by using hygromycin B as selection agent. The vector pHLifeactZtGFP (Fig. 3) contains *ztgfp* fused to the first 17 amino acids of the actin-binding region of ABP140p in *S. cerevisiae* (MGVADLIKKFESISKEE; (Riedl et al., 2008) under the control of constitutive *zttub2* promoter and terminator sequences for random ectopic integration into the genome of *Z. tritici* by using hygromycin B as selection agent. The vector pHeGFPSKL (Fig. 3) contains *egfp* fused to the tripeptide SKL that serve as peroxisome targeting signal under the constitutive *zttub2* promoter and terminator sequences for random ectopic integration into the genome of *Z. tritici* by using hygromycin B as selection agent. The vectors pHmCherrySso1 and pGmCherrySso1 (Fig. 3) contains *mcherry* fused to the full-length *ztsso1* under the control of constitutive *zttub2* promoter and terminator sequences for random ectopic integration into the genome of *Z. tritici* by using hygromycin and G418 as selection agents respectively.

A 14,043 bp fragment of pCeGFPAtg8 (digested with *Bam*HI and *Bgl*II), a 14,611 bp fragment of pCeGFPSso1 (digested with *Bam*HI and *Bgl*II), a 13,597 bp fragment of pCCal^s^eGFPHDEL (digested with *Bam*HI and *Bgl*II), a 14,769 bp fragment of pCHis1ZtGFP (digested with *Bam*HI and *Bgl*II), a 14,916 bp fragment of pCAcd1ZtGFP (digested with *Bam*HI and *Bgl*II), a 13,585 bp fragment of pCLifeactZtGFP (digested with *Bam*HI and *Bgl*II) and a 13,543 bp fragment of pCeGFPSKL (digested with *Bam*HI and *Bgl*II), were individually recombined with 1806 bp hygromycin resistance cassette (amplified with amplified with SK-Sep-128 and SK-Sep-129; Table 2) in yeast *S. cerevisiae* to obtain the vectors pHeGFPAtg8, pHeGFPSso1, pHCal^s^eGFPHDEL, pHHis1ZtGFP, pHAcd1ZtGFP, pHLifeactZtGFP and pHeGFPSKL respectively (Fig. 3). A 14,609 bp fragment of pCmCherrySso1 (Fig. 2; digested with *Bam*HI and *Bgl*II) was recombined either with 1806 bp hygromycin resistance cassette (amplified with primers SK-Sep-128 and SK-Sep-129; Table 2) or 1424 bp G418 resistance cassette (amplified with primers SK-Sep-415 and SK-Sep-416; Table 2) resulting in pHmCherrySso1 and pGmCherrySso1 (Fig. 3) respectively. Note that all these vectors were derived from carboxin resistance conferring vectors (Fig. 2) and as such it contains part of the succinate dehydrogenase gene, carrying the mutation H267L and succinate dehydrogenase terminator. However, these fragments are of no significance. Further details on vector construction and yeast recombination-based cloning is provided in (Kilaru and Steinberg, 2015).

### 4.6 *Z. tritici* transformation and molecular analysis of transformants

The vectors pCeGFPAtg8, pCeGFPSso1, pCCal^s^eGFPHDEL, pCHis1ZtGFP, pCAcd1ZtGFP, pCLifeactZtGFP, pCeGFPSKL and pGmCherrySso1 were transformed into *A. tumefaciens* strain EHA105 by heat shock method (Holsters et al., 1978). *A. tumefaciens* mediated transformation *of Z. tritici* was performed as described previously (Zwiers and De Waard, 2001) with slight modifications. To confirm the integration of vector in to the *sdi1* locus of *Z. tritici* and also to determine the copy number, Southern blot hybridizations were performed by using the standard procedures (Sambrook and Russell, 2001). 3 µg of genomic DNA of IPO323 that exhibited fluorescent signals was digested with *Bgl*II and separated on a 1.0% (w/v) agarose gel and capillary transferred to a Hybond N_ membrane (Amersham Pharmacia Biotech, Little Chalfont, UK). 1014 bp *sdi1* probe (3′ end of the *sdi1^R^* gene and *sdi1* terminator) was generated by with primers SK-Sep-10 and SK-Sep-13 (Table 2) using DIG labelling PCR mix (Life Science Technologies, Paisley, UK). Hybridizations were performed at 62 °C for overnight and autoradiographs were developed after an appropriate time period.

### 4.7 Epi-fluorescence microscopy

Fluorescence microscopy was performed as previously described (Schuster et al., 2011). In brief, cells were inoculated in YG media and grown at 18 °C with 200 rpm for 24 h and placed onto a 2% (w/v) agar cushion for direct observation using a motorized inverted microscope (IX81; Olympus, Hamburg, Germany), equipped with a PlanApo 100×/1.45 Oil TIRF (Olympus, Hamburg, Germany). The various organelles were excited using a VS-LMS4 Laser Merge System with solid-state lasers (488 nm 75 mW and 561 nm 75 mW; Visitron Systems, Puchheim, Germany). Z stacks were generated by using an objective piezo (Piezosystem Jena GmbH, Jena, Germany). Synchronized observation of red and green fluorescence was performed using a dual imager (Dual-View 2 Multichannel Imaging System; Photometrics, Tucson, USA) equipped with a dual-line beam splitter (z491/561; Chroma Technology Corp., Bellows Falls, USA), with an emission beam splitter (565 DCXR; Chroma Technology Corp., Bellows Falls, USA), an ET-Band pass 525/50 (Chroma Technology Corp., Bellows Falls, USA), and a single band pass filter (BrightLine HC 617/73; Semrock, New York, USA). Laser dissection was done using a 405 nm/60 mW diode laser which was coupled into the light path by a OSI-IX 71 adaptor (Visitron System, Puchheim, Germany) and controlled by a UGA-40 controller (Rapp OptoElectronic GmbH, Hamburg, Germany) and a VisiFRAP 2D FRAP control software for Meta Series 7.5.x (Visitron System, Puchheim, Germany). Images were captured using a CoolSNAP HQ2 camera (Photometrics/Roper Scientific, Tucson, USA). All parts of the system were under the control of the software package MetaMorph (Molecular Devices, Wokingham, UK).

### 4.8 Visualization of the plasma membrane

To visualize the plasma membrane marker Sso1 cells of strain IPO323_eGFPSso1 were excited with 20% output power of the 488 nm laser and a single image was taken with 150 ms exposure time. In addition a DIC image was taken for each cell. To localize GFPSso1 with the endocytic marker dye FM4-64 (Molecular Probes/Invitrogen, Paisley, UK), cells were first incubated in YG media containing 100 μM carbonyl cyanide m-chlorophenyl-hydrazone (CCCP; Sigma-Aldrich Chemie Gmbh, Munich, Germany) for 15 min, 18 °C with 200 rpm, followed by an additional 15 min incubation in YG media containing 100 μM CCCP and 1 μM FM4-64 at 18 °C with 200 rpm. The cells were washed by centrifugation for 5 min at 5000 rpm and re-suspended in fresh YG media. Cells were directly placed onto a 2% (w/v) agar cushion and observed using the dual-line beam splitter with 150 ms exposure time, the 488 nm at 50% and the 561 nm laser at 20%. In addition a bright field image was taken for each cell. Overlays of the fluorescent and bright field images were generated using MetaMorph (Molecular Devices, Wokingham, UK).

To visualize the nucleus, endoplasmic reticulum, actin cytoskeleton, mitochondria, peroxisomes and autophagosomes, *Z. tritici* cells expressing His1-ZtGFP, Cal^s^-eGFP-HDEL, Lifeact-ZtGFP, ZtAcd1-ZtGFP, eGFP-SKL and eGFP-Atg8 were imaged in z stacks with a z resolution of 0.2–0.3 μm and an exposure time of 100–150 ms. The 488 nm laser was used at 5% to 50% output power. The final images are maximum projections generated in MetaMorph (Molecular Devices, Wokingham, UK). In addition, a bright field image was taken for each cell.

### 4.9 Nucleus staining using DAPI

The nucleus was counterstained in *Z. tritici* cells expressing His1-ZtGFP and Cal^s^-eGFP-HDEL. Cells were grown in YG media at 18 °C with 200 rpm for 24 h and fixed with 3% (v/v) formaldehyde (Polysciences Europe GmbH, Hirschberg, Germany) for 15 min followed by staining with 0.5 µg/ml DAPI (Sigma-Aldrich Chemie Gmbh, Munich, Germany) for 10 min. Stained cells were placed onto a 2% (w/v) agar cushion and imaged using the DAPI filter set and the standard mercury burner.

### 4.10 Actin cytoskeleton disruption with Latrunculin A

The actin cytoskeleton was disrupted by incubating *Z. tritici* cells expressing Lifeact-ZtGFP in YG media containing 10 µM Latrunculin A (Molecular Probes/Invitrogen, Paisley, UK) for 45 min at 18 °C with 200 rpm. Treated cells were placed onto a 2% (w/v) agar cushion containing 10 µM Latrunculin A, followed by microscopic observation, using an exposure time of 150 ms and a 488 nm laser at 50% output power.

### 4.11 Rhodamine 123 staining

To confirm the correct targeting cells labelled with ZtAcd1-ZtGFP were counterstained with 1 µM Rhodamine 123 (Sigma-Aldrich Chemie GmbH, Munich, Germany) for 30 min at room temperature. Stained cells were placed onto a 2% (w/v) agar cushion and z stacks with a z resolution of 0.3 μm and an exposure time of 150 ms using an objective piezo (Piezosystem Jena GmbH, Jena, Germany) were acquired using a dual imager (Dual-View 2 Multichannel Imaging System; Photometrics, Tucson, USA).

### 4.12 Induction of peroxisome proliferation by oleic acid

IPO323-eGFP-SKL cells were grown in YG media at 18 °C with 200 rpm for 24 h. Cells were centrifuged at 5000 rpm for 5 min and washed twice with nitrogen minimal medium (NM). The pellet was re-suspended in NM medium and equal volumes were transferred into flasks with NM medium containing 1% (w/v) glucose (control) or 1% (v/v) oleic acid (Merck, Darmstadt, Germany) and incubated for 3 h at 18 °C and 200 rpm. Peroxisome numbers in the first 20 μm of the fungal hyphae were determined in maximum projections, generated from Z-axis image stacks in MetaMorph (Molecular Devices, Wokingham, UK) with a z-axis resolution of 0.2 μm and an exposure time of 100 ms and acquired using an objective piezo (Piezosystem Jena GmbH, Jena, Germany).

### 4.13 Induction of autophagy using the mTor-inhibitor rapamycin

IPO323-eGFP-Atg8 cells were grown in YG media at 18 °C with 200 rpm for 24 h. To inhibit the Set/Thr protein kinase mTOR and induce autophagy, cells were treated with 200 nM rapamycin (Selleckchem, Munich, Germany) for 4 h. The number of autophagosomes was analysed in maximum projection generated in MetaMorph (Molecular Devices, Wokingham, UK) from z-axis image stacks at a z-axis resolution of 0.2 μm and an exposure time of 100 ms that were acquired using an objective piezo device (Piezosystem Jena GmbH, Jena, Germany). The cell dimensions in multi cellular structures of *Z. tritici* were determined in bright field images.

## Appendix A Supplementary material

Supplementary video 1 3D-reconstruction of Lifeact-ZtGFP-labelled F-actin in *Z. tritici*. Actin cables concentrate near the multiple growth points. In addition, actin patches appear at the periphery of the multi-cellular macropycnidiospore. The scale bar represents 10 μm.

Supplementary video 2 3D-reconstruction of Lifeact-ZtGFP-labelled F-actin in *Z. tritici*. Actin cables and patches concentrate at the site of septum formation. The scale bar represents 10 μm.
